# Developing a Pocket Park Prescription Program for Human Restoration: An Approach That Encourages Both People and the Environment

**DOI:** 10.3390/ijerph20176642

**Published:** 2023-08-24

**Authors:** Yuting Yin, Yuhan Shao, Yifan Wang, Liuxi Wu

**Affiliations:** 1 College of Architecture and Urban Planning, Tongji University, Shanghai 200092, China; yyin0326@tongji.edu.cn (Y.Y.);; 2 Department of Landscape Architecture, University of Sheffield, Sheffield S10 2TN, UK

**Keywords:** restoration, pocket parks, Park Prescription Program, behavioral pattern, landscape feature

## Abstract

Healing through nature has long been confirmed as an efficient way to improve human physical and psychological health in contemporary urban life. This concept evolved into the well-known Park Prescription Program. However, the psychological restoration imparted by nature was not particularly emphasized in the original Park Prescription Program; it primarily addresses the regulation of physical activities. The quality of urban parks may affect how well people pursue these prescriptions, but the program rarely includes designers among its stakeholders. This study is inspired by the Park Prescription Program, and its intent is to develop a Pocket Park Prescription Program that encourages usage by active people and proper landscape design. The inclusion of designers has been found to be extremely important for pocket parks since they are limited in size but have the advantages of high flexibility and accessibility, and their restorative potential needs be maximized with the limited resources available. Ten pocket parks with distinct functional and landscape attributes were selected in Shanghai as research sites. The Restorative Component Scale was designed into a questionnaire-based survey to measure how people perceived restorative experiences in each site. The onsite survey also incorporated questions investigating people’s behaviour characteristics of using these pocket parks. Site photos were taken and analysed with semantic image segmentation to indicate the landscape compositions of each site. The relations between people perceived restorativeness and parks’ using patterns and landscape characteristics were then explored with correlation analysis to provide cues on instructing how people’s visiting behavior and park landscape design can be improved. The results suggest there are better restoration results when people stay longer in pocket parks, and when people visit their neighborhood pocket parks two to three times a week, these benefits are further enhanced. This study also found that when these prescribed health behaviors are uncertain, the restorative experiences perceived by people can be improved with design interventions in regard to landscape elements such as vegetation, person, decorative lamps, pavement and terrain. These interventions should be made also in consideration of specific park functions. The research outcome intends to show that designers should be included as stakeholders in the Pocket Park Prescription Program, and it is expected to guide individuals towards effectively using pocket parks for restoration. This is to ensure that both the design and the people’s perspectives will be strengthened through the implementation of this program.

## 1. Introduction

The construction of pocket parks has been advocated in Chinese high-density cities as a solution to balancing between people’s increasing needs for urban green spaces (UGSs) and the scarcity of spaces. An important aspect of a pocket park is to bring people health benefits in their everyday urban life, but its psychological influences and restoration have not been particularly emphasized. Referring to the Park Prescription Program, this study brings about a Pocket Park Prescription Program that involves both the users and the designers’ perspectives, so that people can have restorative experiences as efficiently as possible with the limited size of green spaces and limited time of contact with green spaces available.

### 1.1. Park Prescription Program and the Potential of Pocket Parks

Contact with nature can improve the psychological and physical health of human beings with no side effects. Since nature is readily available at a low cost, it becomes a major medical alternative [1]. The Park Prescription Program was first proposed in America in 2009. The National Recreation and Park Association (NRPA) widely promoted the program in 2013 by encouraging people to spend time in nature to improve their health and well-being. Collaborations between park and public land agencies, healthcare providers and community partners were often included [2]. Park prescription is defined as ‘a focus on programs or interventions that … include a health or social service provider, who encourages their patients/clients to spend time in nature and with the goal of improving their health and well-being’ [3]. It was successfully promoted due to America’s previous publication of the Recreation Opportunity Spectrum, which is a multi-scale system for utilizing urban green spaces; it ensures that park prescriptions given by healthcare providers can be easily realized. The NRPA has also established a comprehensive interactive database that records the spatial information of UGSs and recreational facilities [4]. The Park Prescription Program has now been applied in Canada, the UK and other countries around the world. By highlighting the connection between physical activity and chronic disease prevention, most park prescriptions seek to regulate people’s physical activities in parks [5,6]. This includes the length of their stays, their visiting frequency and their exercise activities. However, limited provision of, and access to, wild nature and large-scale parks in highly densified urban environments has forced researchers and practitioners to explore easily accessible, everyday alternatives [7,8,9], for example, pocket parks.

The pocket park concept was derived from the European experience after the Second World War, but it was formalized in America by Robert Zion, who called them ‘vest-pocket parks’ and designed the first pocket park, Paley Park, in 1963 [10]. The theoretical exploration of pocket parks began about 20 years after their practical application [11]; the increase in pocket park research occurred after 2010 when people began to understand the importance of pocket parks in urbanized areas. Pocket parks are distinguished from traditional parks by their small sizes, discrete distribution, flexible site selections, high utilization rates and convenient accessibility [12]. The tiny size of pocket parks allows them to be easily placed in vacant properties scattered throughout the urban fabric [13]. Although the definition of pocket parks ranges from 50–100 feet [14] to 400–10,000 square meters [15] in different countries, their designated primary health-promoting uses are consistently summarized as ‘rest and restitution’ and ‘socializing’ [16]. The promotion of pocket parks began late in China, but the theories and procedures have developed rapidly with the encouragement of national policy since 2014. Earlier relevant studies in China mainly concern pocket parks as components of green infrastructures and concentrate on supply-based ecosystem services such as climate adaptation [17,18], storm control [19,20], urban heat island effect mitigation [21,22], air quality improvement [23] and wind environment improvement [24]. With the ‘Healthy Cities Movements’ [25] and the increasing percentage of urban residents suffering from chronic physical and psychological diseases, scholars soon realized that an important aspect of pocket park services is to introduce people to restorative experiences and encourage them to have outdoor social connections. 

Branching out from the environmental affordance and vitality perspectives, a large measure of the relevant studies focus on exploring social factors that may influence people’s preferences to visit pocket parks, such as companionship [26]; social support and encouragement from families and friends [27]; personal factors of age, gender, education and ethnicity [28]; physical factors of lighting, facilities, seating areas [29] and water features [14]; and perceptual factors of safety, cleanliness and opportunities for gathering [30]. The solid confidence in the health benefits of nature and natural-dominated settings has made people emphasize the social capacity of pocket parks, while their restorative aspect has been relatively overlooked. Moreover, though small-scale green solutions enable people to cope with the shortage of land resources and the time they spend in fast-paced, modern urban life, they are small in size and have limitations in terms of encouraging long staying behavior in users which can provide them with evident health improvements. This may also explain the insufficient attention being paid to pocket parks in the Park Prescription Program. Previous evidence suggested that even limited amounts of time spent in nature can accumulatively play an important role in effective functioning and well-being [31], thereby contributing to the restoration process of pocket park users. Therefore, it is believed that the attempt to explore and increase restorative potential within commonly available and routinely encountered pocket parks though a design perspective is necessary and important, as pocket park restorative experiences, however fleeting, are more easily and frequently accessible than a daytrip away from a city, staying in a remote natural environment or spending a few hours wandering in an urban park. A perspective concerning both human health behavior and restorative environment design should be introduced to maximize the perceived restorative experiences of people in pocket parks, so as to adapt to the increasing importance of pocket parks in forming a more applicable Park Prescription Program.

### 1.2. The Restorative Benefits of Urban Nature and Pocket Parks

Natural environments have long been deemed to have restorative benefits to human health. This view can be traced as far back as the earliest ‘healing gardens’ [32] that were formerly popular in health care institutions to aid patient recovery. The investigation regarding positive interactions between humans and nature has mainly progressed in the domain of environmental psychology, with one branch gradually evolving into restorative environment research. However, this widely adopted awareness was not formalized until 1984, when the first report about the measurable effects of nature’s influence on health was published [33]. The restoration process people can undergo when exposed to a natural environment is the underlying reason why restorative environment research was first promoted. Four importance attributes of nature, being away, extent, fascination and compatibility can help people to attain (1) tranquility, peace and silence, which human beings require at least occasionally, (2) integration and wholeness, which lead to a significant life development goal and improved self-esteem and (3) oneness, which is a sense of being at one with the universe. These characteristics and recovery effects of nature were then developed into Kaplan and Kaplan’s attention restoration theory (ART) [11,34,35,36] that mainly deals with the renewal of a depleted capacity for directing or focusing one’s attention. 

Though the urbanization process has significantly decreased opportunities for urban residents to encounter nature, there is no reason to believe that urban settings lack these restorative attributes that natural environments have. Thus, a shift in the research which focuses on restorative environments was facilitated from nature to urban nature. The restorative benefits of a park, a typical urban natural space, have been validated in relevant studies. It was found that just a glance at a small park on the way to work might have a positive influence on mood [37]. Viewing a nature-dominated park through a window at home or in the workplace can support micro-restorative experiences [38,39]. Ekkekakis et al. [40] found that travelling through a park setting for a brief 10–15 min may provide a respite that interrupts the process of resource depletion and promotes a shift towards increased activation and positive moods [41,42]. This benefit has also been proven to be effective for the elderly [43] and children [44]. Researchers subsequently noted that environments with certain prominent natural characteristics can enable people to recover their mental and physical resources when they have been depleted in stressful or wearying situations [45]. Other research has found that urban parks are closely correlated with restoration likelihoods and lead to perceived stress recovery, enhanced attention restoration and positive physiological responses [46,47,48]. This is also the result when trees, flower beds and other natural elements are present along residential streets [49,50] and when natural components are present in pocket parks within neighborhoods [51,52].

The restorative benefits of pocket parks can be traced back to the early 1980s [11] and then systematically explored in a series of studies conducted by Nordh and his associates [7,46,51]. Nordh et al. [51] claim that a person is more likely to experience restoration in larger parks; however, some of the smallest parks in their sample show some of the highest restorative value ratings. This finding was then proven by another study showing that the most stressed users receive stronger feelings of being removed from everyday pressure and obligations when nature is present in small parks [16]. Using interviews [7,46], Photoshop grid analysis [51] and eye tracking [52], researchers have also explored physical factors contributing to the restorative benefits of pocket parks. They found that grass, flowers, bushes and water features are the park components that are most likely to promote restoration. Peschardt et al. [16] investigated the landscape features of pocket parks relative to two major health promoting uses in particular and found obvious differences. Their results indicate that green features do not seem to be of crucial importance for socializing, whereas features that promote gathering should be prioritized. For the purposes of rest and restitution, the main results show that green ground cover and enclosed green niches are important, while disturbing features (e.g., playground, view outside park) should be avoided [16]. However, the existing evidence either investigates people’s restorative perceptions in pocket parks or focuses on certain restorative contributors. The synergistic relationships between multiple factors have been neglected. There is no coherent approach that informs how the design of specific environmental attributes can help in achieving restoration and other health objectives; this also limits the ability to sustain restorative roles in practical applications.

### 1.3. This Study

Inspired by the Park Prescription Program and the different paths required to achieve diverse health objectives from pocket parks, this study intends to develop a Pocket Park Prescription Program that aims at exploring and enhancing restorative benefits people can have when using pocket parks. It is also proposed that in a Pocket Park Prescription Program, the increased use of pocket parks and the individuals who have obtained psychological benefits during their visits are equally important. Thus, attention must be given to both the research and the design procedures.

The restorative component scale (RCS) [53], a widely used psychometric to measure people’s restorative experiences, was designed into a questionnaire-based survey with questions investigating people’s behaviour patterns for using pocket parks. Landscape characteristics of pocket parks were analysed with semantic image segmentation, a technique developed based on Artificial Intelligence. The relations between people perceived restorativeness and parks’ using patterns and landscape characteristics were then explored with correlation analysis to provide cues on instructing how people’s visiting behavior and park landscape design can be improved.

## 2. Method

### 2.1. Research Sites

The regeneration and construction of pocket parks is an important part of the Park City agenda advocated by the city of Shanghai. It is also an efficient means of coping with the shortage of green spaces in highly dense megacities. In 2022, 130 pocket parks had been completed and were open to the public in Shanghai; this number is expected to reach 300 by 2025. However, the current Technical Guidelines for the Construction of Pocket Parks in Shanghai only address the construction and acceptance standards without considering the restorative design requirements. Shanghai is a suitable city for this study due to its strong development intention and its insufficient practical considerations. 

Pocket parks were selected based on following criteria: (1) they should be located within the inner ring road of Shanghai where the city population aggregates; (2) they should be 2000–5000 square meters in size referring to the size defined for Chinese pocket parks [15]. Also, the differences in size may also influence people perceived restorativeness [7]; (3) they should cover three major pocket park types: landscape and aesthetics, living and services and exercise and fitness. Ten pocket parks were determined as case study sites; two of them are in the Huangpu district, two of them are in the Jing’an district, two of them are in the Hongkou district, two of them are in the Yangpu district and two of them are in the Pudong New district. Four pocket parks mainly carry landscape and aesthetics functions, five are designed for living and services purposes and one has exercise and fitness facilities (Table 1).

### 2.2. Measurements

In response to the aim of this study, two parallel methods of investigation were conducted. A questionnaire-based onsite survey was used to obtain people’s restorative perceptions in case study sites and their regular behaviour patterns in using them, while semantic image segmentation was used to obtain the landscape characteristics of the sites. People’s restorative perceptions and use patterns were then mathematically correlated with parks’ landscape characteristics, so as to explore whether and how parks’ landscape characteristics can have positive benefits.

#### 2.2.1. RCS to Measure Pocket Park Restorativeness

After referring to similar studies [54,55] that were conducted within a Chinese context, a revised version of the RCS [53] that is more conveyable in Chinese was employed to investigate participants’ perceptions of restorations in the ten selected pocket parks. The RCS contains 15 items (Table 2); three of them describe the being away (B) component, three of them describe the extent (E) component, five of them describe the fascination (F) component and four of them describe the compatibility (C) component proposed in the ART [11,34,35,36]. The revised RCS is used as a questionnaire, and the responses for each item are collected based on a 5-point Likert scale (1–5, representing strongly disagree [1] to strongly agree [5]). 

#### 2.2.2. Survey Questions to Investigate People’s Behavioral Attributes

Three questions followed the RCS to investigate the frequency, duration and purposes of visits to these pocket parks. Background information, including gender, age and professional relevance, was also collected from each questionnaire participant to explore whether there were individual perceptive differences. 

#### 2.2.3. Semantic Image Segmentation to Describe Pocket Park Characteristics

**Figure 1:**
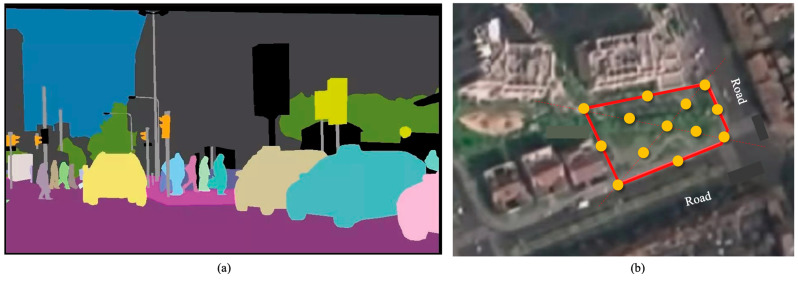
(**a**) Illustration of semantic image segmentation. (**b**) Selection of photography spots.

Semantic image segmentation was used to describe the landscape attributes of the research sites to establish the connection between human restorative perceptions and pocket park characteristics in a comprehensive way. Semantic image segmentation is a technique based on artificial intelligence that enables us to associate each pixel of a digital image with a class label, such as trees, signboards, pedestrians, roads, buildings, cars and the sky (Figure 1a). It has been widely used in studies that need to quantify environmental characteristics from a massive number of photos or videos as visual stimulus, and it works as an efficient alternative to previous subjective assessment and pixel calculation methods.

Since it lacks massive data sources such as street view images for pocket parks, this study took photos of the research sites. To ensure that image data fit the requirements for conducting semantic segmentation, at least nine photography spots should be selected within each site and four photos should be taken in each direction (facing south, facing west, facing east and facing north). Hence, the minimum number of photos for each site should be over 36. Photography spots were determined along the side boundaries and diagonal lines according to the geometric features of the ten pocket parks (Figure 1b). Each photo has a 16:9 scale and a pixel size of 1000 by 1000. All surveyors were trained before they formally took photos of sites.

### 2.3. Data Collection

The RCS questionnaire and behavior questions were edited into an online version using the Wenjuanxing website [see https://www.wjx.cn/ (accessed on 5 May 2023)] before the survey was formally taken, an online open platform that is used for designing, editing and promoting questionnaires and surveys. The on-site questionnaire surveys were conducted on sunny days with an air quality index of less than 60 and a wind speed below 5 m per second throughout May 2023.

Subjects were recruited at each chosen park with the following requirements: (1) they were alone at the park [26], (2) they were between 22 and 55 years of age, (3) they were not in a hurry and (4) they all had normal or corrected-to-normal visual acuity and normal color vision. They were asked if they are willing to participate in a study investigating health benefit of pocket parks and those who had interest were then briefed of the survey procedures by researchers. When subjects clicked on the survey link, they were first shown a brief introduction to the survey and were asked to sign the consent form if they were willing to participate. They were then directed to the park rating page.

The sample size in this study was decided referring to the effect sizes of existing studies on restorative environments. The impact of a restorative environment on subjectively reported fatigue revealed an effect size of 1.28 [56] (minimal sample size = 6), while studies of the impact on cortisol, a biological measure of stress and non-restorativeness, revealed an effect size of 0.57 (minimal sample size = 24) [57]. Hence, the appropriate number of participants should be approximately 10–30. Each subject was asked to experience the site for at least three minutes to give them enough time to become mentally immersed in the setting [58,59] and complete the questionnaire afterwards. A total of 20–30 responses were required for each pocket park during the three workdays allocated. Noise data, such as incomplete questionnaires or questionnaires with the same ratings for every indicator, were manually removed; the number of final valid responses was 232. Of that number, 17 responses were for HP1, 19 were for HP2, 23 were for JA1, 30 were for JA2, 20 were for HK1, 25 were for HK2, 22 were for YP1, 26 each were for YP2 and PD1 and 24 were for PD2.

### 2.4. Data Analysis

The data include the participants’ restorative experiences measured by the RCS in the ten pocket parks, their behavior patterns for visiting the pocket parks and the landscape characteristics of the pocket parks calculated with semantic image segmentation. The RCS results were checked for reliability to see whether they were suitable for further analysis. The results were also tested for potential individual perceptive differences. The restorative benefits of the research sites were then compared using the mean ratings of the overall restorativeness and the four ART components described in the RCS (being away, fascination, extent and compatibility), respectively. Pearson correlation analyses were then conducted to determine whether and how each landscape element identified from site photos and people’s visiting habits influence their perceived restorative benefits in the ten pocket parks. It was regarded as an appropriate analysis method since all variables in this study are continuous variables; in relation to the same individual and have linear relationship referring to their P-P plot.

## 3. Results

### 3.1. Manipulation Checks

Questionnaire responses about the restorative benefits in the ten selected pocket parks were examined for internal consistency with Cronbach’s α using SPSS Statistics V27 software. It is the preferred measurement of inter-rater reliability when cases are rated in terms of an interval variable or interval-like variables [60]. The α values of the assessment results showed sufficient internal consistency (Cronbach’s α > 0.8) and thus proved to be reliable for further analysis. 

Perceptive differences between groups of participants with different backgrounds (gender, age and professionality) were also analyzed using an independent sample *t*-test. The Levene test values of variance equation were all greater than 0.05, and the data were consistent in the homogeneity of variances. The *t*-test significance values of mean equations were also greater than 0.05, which showed that the gender, age and professional backgrounds of participants did not cause significant differences in their restorativeness evaluations.

### 3.2. Descriptive Analysis of the Ten Pocket Parks and the Participant Usage Patterns

#### 3.2.1. Participant Usage Patterns in the Ten Pocket Parks

**Figure 2:**
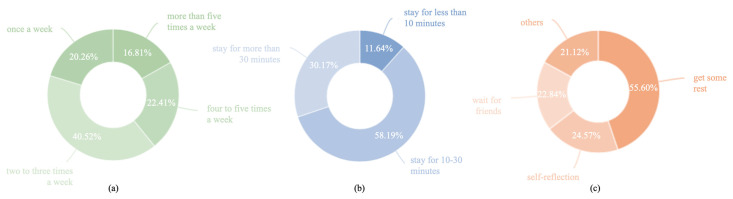
Peoples’ frequencies (**a**), durations (**b**) and purposes (**c**) when visiting pocket parks.

Questionnaire results showed that among the 232 subjects who participated in the study, nearly 80% of them visited pocket parks more than twice a week (two to three times a week 40.52%, four to five times a week 22.41% and more than five times a week 16.81%), and a good number of them visited two to three times a week (40.52%). Individuals who went to pocket parks once a week (20.26%) and four to five times a week (22.41%) were fairly close. Only 16.81% of the 232 participants went to their nearby pocket parks more than five times a week (Figure 2a).

In addition to visiting frequency, staying duration is another important behavioral factor that may influence people’s perceived restorative benefits [61,62]. In general, over 88% of the surveyed participants stayed for more than 10 min every time they visited a pocket park (stay for 10–30 min 58.19% and stay for more than 30 min 30.17%), and the highest percentage was participants who stayed 10–30 min (58.19%). A total of 30.17% of the subjects stayed at the pocket parks for more than 30 min, and those who stayed for less than 10 min accounted for only 11.64% of the total (Figure 2b).

In terms of visiting purposes, more than 55.6% of the subjects stated that they had no particular purpose when they visited a pocket park; they only wanted some rest. Another 24.57% of them indulged in self-reflections and enjoyed their alone time while they were there. Other people (22.84%) chose pocket parks as rendezvous points where they met with their friends. The remaining 21.12% of the subjects included those who were there to exercise, to walk their dogs, to play with children and to play musical instruments (Figure 2c).

#### 3.2.2. Landscape Characteristics of the Ten Pocket Parks

A total of 19 indicators were identified based on labels classified from the street view training dataset; they included road, sidewalk, building, wall, fence, pole, traffic light, traffic sign, vegetation, terrain, sky, person, rider, car, truck, bus, train, motorcycle and bicycle. Considering that pocket parks have slightly different landscape elements, manual corrections were made based on real situations that included combining road and sidewalk into ‘pavement’, combining building and wall into ‘landscape constructions (i.e., resting and exercising facilities, pavilion and veranda)’, changing traffic light to ‘decorative lamp’, changing traffic sign to ‘signage’, and combining car, truck, bus, train, motorcycle and bicycle to ‘surrounding vehicle’.

**Figure 3:**
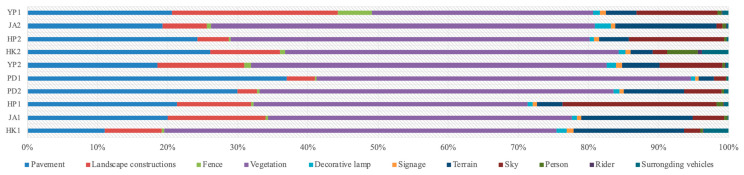
Landscape characteristics of the ten pocket parks measured by semantic segmentation.

The semantic segmentation results showed that vegetation constituted the highest percentage of the landscape composition in all the research sites, ranging from 31.48% (YP1) to 55.92% (HK1). Pavement accounted for 11.04% (HK1)–36.97% (PD1), landscape constructions accounted for 2.77% (PD2)–23.71% (YP1), terrain accounted for 2.15% (PD1)–15.80% (JA1) and sky accounted for 0.82% (JA2)–21.98% (HP1). Following with decorative lamp (accounted for 1.54% in HK1, accounted for 1.32% in YP2, accounted for 2.27% in JA2 and accounted for 1.01% in YP1), person (accounted for 1.01% in HP1 and accounted for 4.27% in HK2) and fence (accounted for 4.83% in YP1) that mostly accounted for less than 1.00%. The percentage of signage and rider are all under 1.00% (Figure 3, Table 3).

Although the general tendencies of the landscape composition appeared to be close, the landscape features of the exercising and fitness category (YP1) varied from the other two types (landscape and aesthetics and living and services). Instead of being dominated by vegetation as the type of landscape and aesthetics (39.05–55.92%) and living and services (50.76–54.70%), vegetation (31.48%), landscape constructions (23.71%) and pavement (20.59%) in the exercising and fitness category are evenly distributed and together constitute the major characteristic of YP1. Sky (11.45%), fence (4.83%) and terrain (4.47%) are also important elements to sustain the exercising and fitness function of this type. 

The ranking order of the landscape elements in the landscape and aesthetics parks (JA2, HP2, HK2 and YP2) and the living and services parks (PD1, PD2, HP1, JA1 and HK1) are quite similar; there are only slight differences in the proportions of the landscape constructions and the terrain. The former type has a larger proportion of landscape constructions (mean = 8.27%), while the latter has more terrain (mean = 9.16%). Moreover, HP2 (13.65%) and YP2 (8.90%) in the landscape and aesthetics type and HP1 (21.98%) in the living and services type contain more sky than terrain (HP2 4.28%, YP2 5.32% and HP1 3.52%), while the other six pocket parks (JA2, HK2, PD1, PD2, JA1 and HK1) have converse characteristics.

**Figure 4:**
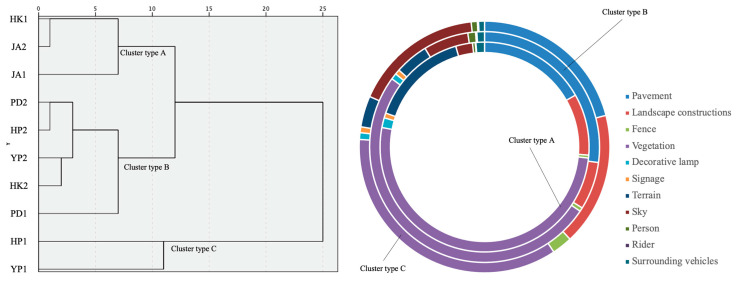
Landscape characteristics of the pocket park types based on hierarchical cluster analysis.

The landscape compositions identified using semantic segmentation were also put through hierarchical cluster analysis to verify their functional categorizations. The cluster results showed that there were only slight differences: HK1, JA1 and JA2 were classified as type A, PD1, PD2, YP2, HK2 and HP2 were classified as type B and HP1 and YP1 were classified as type C. Comparing the compositional features of the three cluster types, it can be observed that cluster types A and B were dominated by vegetation and have similar characteristics with landscape and aesthetics and living and services types, while cluster type C was characterized by sky, landscape constructions and terrain in addition to vegetation that is more consistent with the characteristics of the exercising and fitness category (Figure 4).

#### 3.2.3. Restorative Benefits of the Ten Pocket Parks

**Figure 5:**
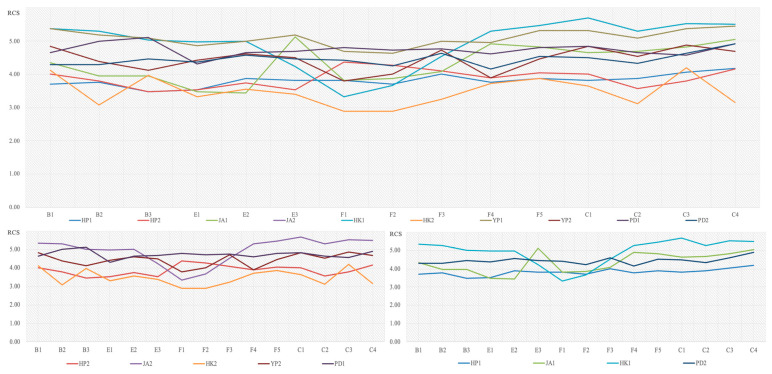
Restorative benefits of the ten pocket parks measured by RCS.

The RCS rating results were calculated and compared with the mean values and line chart. The results indicated that YP1, JA2 and HK1 had the most restorative potential among the ten pocket parks, and HP2, HK2 and HP1 offered the least restorative experiences to users. This tendency also applied to each of the four ART aspects, especially fascination. In terms of the fascination aspect, YP1 (mean = 4.92, SD = 1.01), JA2 (mean = 4.46, SD = 1.14) and PD1 (mean = 4.75, SD = 0.98) were rated as the highest, while HP1 (mean = 3.84, SD = 1.33), HP2 (mean = 4.14, SD = 0.41) and YP2 (mean = 4.12, SD = 0.65) were rated with the fewest fascination opportunities. The highest ratings on the being away aspect appeared in JA2 (mean = 5.23, SD = 0.62), following with YP1 (mean = 5.21, SD = 0.62) and the lowest are HP1 (mean = 3.65, SD = 1.28). For the extent aspect, YP1 (mean = 5.02, SD = 1.02) is also evidently higher than the others and the lowest rating was observed on HK2 (mean = 3.43, SD = 0.60). The compatibility aspect showed a slight difference with the other three, with JA2 (mean = 5.51, SD = 0.66) having the highest rating and HK2 (mean = 3.53, SD = 0.57) the lowest, Figure 5, Table 4. 

Among the three functional types, the exercising and fitness type (YP1) was rated with the highest restorativeness in all of the four ART aspects. The landscape and aesthetics type (HK1, HP1, HK1 and PD2) was better than the living and services type (HP2, JA2, HK2, YP2 and PD1) in terms of delivering the sense of being away, fascination and compatibility, while the five sites of the living and services type were rated higher than the four landscape and aesthetics parks (Figure 5, Table 4).

### 3.3. Landscape and Behavioral Factors in Relation to People’s Perceptions of Restorativeness

#### 3.3.1. The Influences of Landscape Features on People’s Perceptions of Restorativeness

Pearson correlation analysis was then conducted between people’s restorative perceptions and the landscape features of the sites. A total of 15 RCS items and 11 landscape elements were set as variables to explore their mutual influences. The results revealed six park elements that provided users with restorative experiences, among which vegetation and terrain were more likely to have positive effects and person, rider and surrounding vehicles proved to be negatively related. Vegetation showed consistent beneficial influences for all four of the ART aspects, while terrain could only promote the sense of fascination and compatibility. As for negative elements, the presence of others (person) within the site could have hindered people’s restoration processes, since it may impede the senses of being away, fascination and compatibility. Also, rider and surrounding vehicles were found to have negative influences on environmental fascination and compatibility, Table 5.

Influential landscape elements were also explored within the functional groups. In terms of the living and services type, vegetation, person and rider were found with promotive effects towards human restoration, while only surrounding vehicles may have negative influence in this type of pocket park. Less evidence was disclosed for the landscape and aesthetics type; vegetation, decorative lamp, pavement and terrain were shown with positive influences, and no negative elements were observed, Table 5.

#### 3.3.2. The Influences of People’s Usage Patterns on Their Perceived Restorativeness

**Figure 6:**
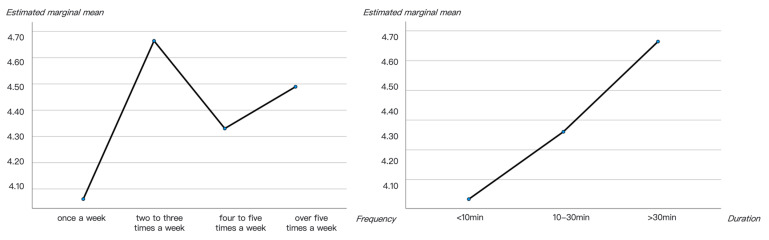
The relationship between visiting frequency and duration and people’s perceived restorativeness.

Pearson correlation analysis was also used to explore whether and how people’s patterns of use of pocket parks influenced their perceived restorativeness. RCS ratings and people’s visiting frequency (once a week was given a value of ‘4’, 2–3 times a week was given a value of ‘3’, 4–5 times a week was given a value of ‘2’ and over 5 times a week was given a value of ‘1’) and duration (less than 30 min was given a value of ‘1’, 10–30 min was given a value of ‘2’ and more than 30 min was given a value of ‘3’) of the 232 responses were set as variables to explore their mutual influences. The results showed that the duration of the stays had an evident linear relationship with the overall restorativeness (average of the 15 RCS items), and it had positive effects on 11 RCS items, excluding B3 (‘I do not need to consider my responsibilities and obligations when I am here’), E2 (‘The existing elements belong here’), F1 (‘There are plenty of things to discover here’) and F2 (‘This setting has many things that I wonder about’). Thus, people who stayed longer in pocket parks had higher restorative perceptions. However, the effect of visiting frequency was inconsistent, and the strongest restoration potential was most likely to occur when people visited pocket parks two to three times a week. The inconsistency also appeared in influencing factors, where promotive effects was only observed only on F1 and inhibitive effects was only observed on C3 (‘I can quickly adapt to this setting’), Table 6, Figure 6.

## 4. Discussion

Pocket parks are proposed in this study as potential substitutes for large-scale city parks in high density cities so people can use nature to heal. Pocket parks have advantages in time and spatial accessibility and become inevitable supplements for urban park systems. However, the restorative benefits of pocket parks may be constrained by their relatively small sizes and the time people spend in them. Existing studies have confirmed positive relationships between park size and people’s perceived restorative benefits [51] and also between the length of people’s stay and their obtained restoration [61,62]. Therefore, a pocket park must maximize its restorative potential using the limited resources that are available, so as to become an essential part in Park Prescription Program. This study asserts that a perspective involving both people’s visiting behaviors and the landscape designs of the pocket parks is necessary for the Pocket Park Prescription Program.

### 4.1. A Dual-Dimensional Pocket Park Prescriptions for Improving Human Restorative Opportunities

Wide literature studies have presented similar conclusions and indicated that living close to nature [9,36], spending time engaged with it or even simply knowing it exists nearby [37] can benefit individual well-being through reduced brain reactivity to stress [63,64], improvements in cognitive and emotional functioning [65] and potentially through facilitating physical recovery from illness [63,66]. However, ‘if the greens are more than three minutes away, the distance overwhelms the need’ [67]; this further highlights the fundamental difficulties between densified urban populations and sufficient provision of urban greenery and the importance of pocket park as a potential solution.

From the perspective of encouraging people’s health behaviors, this study investigated the behaviors of people visiting pocket parks and the influences the parks had towards the restorative benefits attained. The results suggest that health regulations on visiting durations are more important than visiting frequency for restoration purposes. This finding agreed with previous research findings [68]. The longer people stay in pocket parks, the better restoration they can achieve, and if people can visit their nearby pocket parks two to three times a week, these benefits can be further enhanced. 

From the perspective of excavating restorative potential of pocket parks, this study found that when these prescribed health behaviors cannot be guaranteed due to a change in a business schedule or the weather conditions, people’s perceived restorative experiences can still be strengthened through landscape design interventions that may include (1) increasing the use of vegetation that is regularly trimmed and maintained, (2) designing terrain such as slopes, steps and artificial hills to provide people with a stronger sense of fascination, as well as sufficient resting spaces, if allowed, (3) considering designs that occlude sight along the park boundaries to ensure a sense of being away, (4) emphasizing the hierarchy of social distances in design so that people can freely choose their preferred means of mental recovery and (5) placing soft barriers between park users and passers-by at the entrances of pocket parks to avoid mutual interferences. These instructions for park users’ health behaviors and for landscape design, together with collaborations between parks and public land agencies, would constitute the entire picture of the Pocket Park Prescription Program proposed in this study.

### 4.2. Restorative Design Instructions for Different Functions of Pocket Parks

One of the major limitations of delivering healthy parks is the lack of a coherent approach that can efficiently inform how the design of specific environmental attributes can help in achieving attention restoration and other health objectives. The root of the concept of restorative environments in environmental psychology makes it a difficult notion to convey, not only to the average person, but also to urban design professionals committed to enhancing environmental restorativeness. Previous studies can either only measure people’s restorative perceptions in parks that vary in size, function and category (forest parks, city parks and pocket parks) or identify restorative-related park elements in a time-consuming and inefficient (i.e., questionnaire, interview) way. This study explores an innovative way of bridging the gap between human perceptions and environmental attributes through incorporating traditional RCS measures with semantic image segmentation.

Kaplan and Kaplan [36] and other scholars have proposed that to achieve expert judgements on the value of a setting, it is necessary to make a series of decisions regarding the categories to which it belongs and who will use it. An individual’s evaluation of a certain place is closely related to whether their ‘purposes’ are fulfilled when they stay in that place. Therefore, this study also proposes that when the design instructions are applied to specific pocket parks, their functional features should also be considered. According to the correlation analysis results, the restorative contributors vary with the park functions. The positive effect of vegetation is dominant for pocket parks with the living and services function. Control over the occlusion of sight through soft landscapes is also necessary for this type. In terms of the landscape and aesthetics type of pocket park, no negative clue was observed in this research, but the promotive design of vegetation, decorative lamps, pavement and terrain would efficiently optimize the restorative benefits of this type of pocket park. Thus, restorative design instructions for these pocket parks should include (1) increasing the use of well-designed vegetation and dedicated decorative lamps to attract people and immerse them in these settings, (2) involving terrain to encourage people’s staying behaviors and lengthen their visiting durations and (3) using pavement that is adaptable to its surrounding contexts and paying extra attention to its regular maintenance and refurbishment. Due to the limited samples in the exercising and fitness type of park, design differences can only be differentiated between the living and services type and the landscape and aesthetics type. However, it is reasonable to assume that landscape elements in relation to the restorativeness of exercising and fitness pocket parks are different from the other two types of parks, because the landscape compositions in the exercising and fitness type are different from the other two types of parks. These differences are also reversely proved in hierarchical analysis.

### 4.3. Limitations

Although the research findings meet with the study purposes and no obvious contrast was found between the outcomes of this study and previous research, there were limitations relative to the experimental design and data analysis processes. First, only one pocket park belongs to the exercising and fitness type within the ten case study sites. The quantitative imbalance between the three functional types is caused by their inherent unequal provision in Shanghai. Although people’s restorative perceptions were measured on a 5-point Likert scale and their behavior characteristics were described using values of 1–5, the landscape components of the study sites were represented with a percentage. Thus, the differences in a component’s order of magnitudes could influence the correlation results. In addition, this study used a training dataset and algorithm that was originally developed for street view images in the semantic segmentation due to the lack of appropriate images for pocket parks. Artificial amendments were made to redefine the pixel labels, but the accuracy of the landscape characteristic analysis results might still be influenced because of these technological constraints. Another issue relating to the application of semantic segmentation in this study is that the results can only indicate landscape components without showing attributes describing environmental quality, such as enclosure, visual depth and complexity. The overall results are considered to be useful, since the relationship between environmental quality and landscape components has been well established. It is also deemed that the relationship between human perceptions and environmental attributes can be efficiently established using relevant instruments and techniques that will be developed in the future.

## 5. Conclusions

The Park Prescription Program places its primary emphasis on people’s physical activities rather than the psychological restoration of nature. However, restoration was found to be an extremely important function of pocket parks, since they are limited in size but have the advantages of high flexibility and accessibility. This study proposes that physical uses and the psychological benefits that people obtain during their visits are equally important in pocket parks. This requires research and design attention. Therefore, a Pocket Park Prescription Program that encourages usage by active people and proper landscape design is developed in this study through the investigation of usage behaviors, perceived restorativeness and landscape characteristics of Shanghai pocket parks—as well as their mutual influences. The research outcome is expected to instruct healthcare givers to efficiently include restoration in pocket parks, and to include designers as stakeholders in the Pocket Park Prescription Program. Their inclusion will ensure that this program’s implementation is strengthened in both the design and people perspectives. Design instructions developed in this study can also become supplementary materials for the current Technical Guidelines for the Construction of Pocket Parks in Shanghai.

## Figures and Tables

**Table 1:** General descriptions of the selected ten pocket parks.

No.	Location	Site	Size (m^2^)	Function	Site Photo
1	Huangpu District	Zhuimeng Garden (HP1)	5000	Landscape and aesthetics	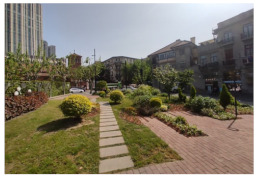
2		Landiao Garden (HP2)	3594	Living and services	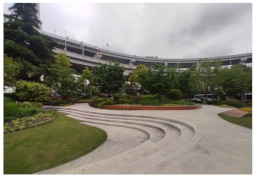
3	Jing’an District	Jinxiu Garden (JA1)	4359	Landscape and aesthetics	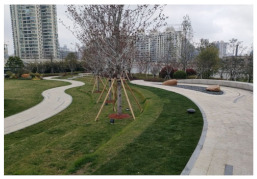
4		Zhijiang Garden (JA2)	4206	Living and services	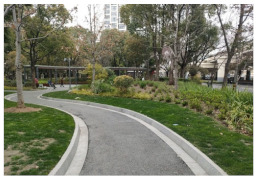
5	Hongkou District	Beiwaitan City Garden (HK1)	2824	Landscape and aesthetics	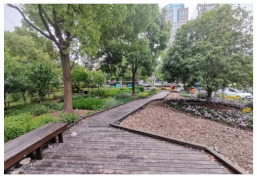
6		Xiahaimiao Garden (HK2)	2030	Living and services	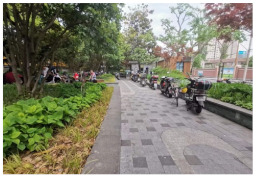
7	Yangpu District	Magic Garden (YP1)	3500	Exercise and fitness	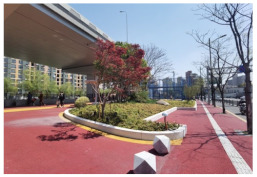
8		Honglan Garden (YP2)	4450	Living and services	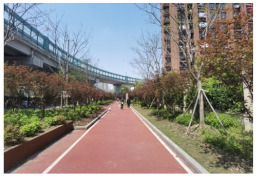
9	Pudong New District	Koelreuteria Paniculata Garden (PD1)	3615	Living and services	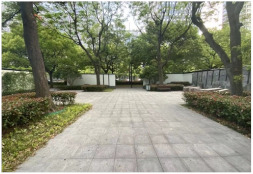
10		Ancient Plum Garden (PD2)	3750	Landscape and aesthetics	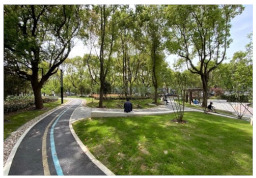

**Table 2:** The revised RCS based on the work of Laumann and his associates [53].

ART Components	RCS Statement
Being away (B)	B1: I feel free from work and my daily routine when I am here. B2: I feel free from other people’s demands and expectations when I am here. B3: I do not need to consider my responsibilities and obligations when I am here.
Extent (E)	E1: The elements presented here go together. E2: The existing elements belong here. E3: The surroundings are coherent.
Fascination (F)	F1: There are plenty of things to discover here. F2: This setting has many things that I wonder about. F3: There are many objects here that attract my attention. F4: There are plenty of things that I want to linger with here. F5: I am absorbed in these surroundings.
Compatibility (C)	C1: The environment allows me to do activities I like. C2: I can handle the kinds of problems that arise here. C3: I can quickly adapt to this setting. C4: There is an accordance between what I like to do and this environment.

**Table 3:** Percentage of landscape elements in each of the ten pocket parks.

	Site No.	Pavement	Landscape Constructions	Fence	Vegetation	Decorative Lamp	Signage	Terrain	Sky	Person	Rider	Surrongding Vehicles
Landscape and aesthetics	HK1	11.04%	8.09%	0.36%	55.92%	1.54%	0.90%	15.74%	2.40%	0.28%	0.16%	3.57%
	JA1	20.02%	13.96%	0.36%	43.26%	0.72%	0.68%	15.80%	4.56%	0.46%	0.06%	0.11%
	HP1	21.31%	10.52%	0.41%	39.05%	0.73%	0.70%	3.52%	21.98%	1.01%	0.15%	0.62%
	PD2	29.93%	2.77%	0.37%	50.51%	0.84%	0.67%	8.61%	5.28%	0.34%	0.08%	0.60%
Living and services	PD1	36.97%	3.99%	0.25%	53.40%	0.56%	0.49%	2.15%	1.75%	0.10%	0.04%	0.27%
	YP2	18.53%	12.36%	0.96%	50.76%	1.32%	0.89%	5.32%	8.90%	0.39%	0.07%	0.46%
	HK2	26.05%	9.86%	0.79%	47.45%	0.97%	0.76%	3.19%	2.05%	4.27%	0.66%	3.80%
	HP2	24.24%	4.50%	0.28%	51.20%	0.59%	0.66%	4.28%	13.65%	0.31%	0.05%	0.24%
	JA2	19.25%	6.35%	0.62%	54.70%	2.27%	0.67%	14.38%	0.82%	0.55%	0.06%	0.31%
Exercising and fitness	YP1	20.59%	23.71%	4.83%	31.48%	1.01%	0.80%	4.47%	11.45%	0.58%	0.15%	0.91%
Mean		22.79%	9.61%	0.92%	47.77%	1.06%	0.72%	7.75%	7.29%	0.83%	0.15%	1.09%

**Table 4:** RCS ratings of the ten pocket parks.

		B1	B2	B3	B-M	E1	E2	E3	E-M	F1	F2	F3	F4	F5	F-M	C1	C2	C3	C4	C-M
HP1	Mean	3.71	3.76	3.47	3.65	3.53	3.88	3.82	3.74	3.82	3.71	4.00	3.76	3.88	3.84	3.82	3.88	4.06	4.18	3.99
	Std. Dev	1.21	1.44	1.46	1.28	1.23	1.45	1.42	1.20	1.51	1.45	1.46	1.52	1.41	1.33	1.38	1.50	1.43	1.42	1.35
HP2	Mean	4.00	3.79	3.47	3.75	3.53	3.74	3.53	3.60	4.37	4.26	4.11	3.89	4.05	4.14	4.00	3.58	3.79	4.16	3.88
	Std. Dev	0.47	0.79	1.07	0.46	1.07	1.10	0.96	0.76	0.60	0.73	0.88	0.74	0.62	0.41	0.75	0.84	0.85	0.83	0.46
HP1	Mean	4.35	3.96	3.96	4.09	3.48	3.43	5.13	4.02	3.83	3.87	4.09	4.91	4.83	4.30	4.65	4.70	4.83	5.04	4.80
	Std. Dev	0.65	0.82	0.88	0.68	0.95	0.84	0.76	0.69	0.98	1.01	0.85	0.85	0.94	0.72	0.83	0.70	0.83	0.93	0.62
JA2	Mean	5.37	5.30	5.03	5.23	4.97	5.00	4.23	4.73	3.33	3.67	4.53	5.30	5.47	4.46	5.70	5.30	5.53	5.50	5.51
	Std. Dev	0.61	0.65	1.10	0.62	0.76	1.14	1.17	0.72	1.52	1.56	1.50	1.34	1.04	1.14	0.60	1.09	0.78	1.01	0.66
HK1	Mean	5.37	5.30	5.03	4.08	4.97	5.00	4.23	4.40	3.33	3.67	4.53	5.30	5.47	4.28	5.70	5.30	5.53	5.50	4.35
	Std. Dev	0.99	1.38	1.57	0.98	1.14	1.29	1.33	1.02	1.43	1.43	1.01	1.38	1.29	0.83	1.47	1.45	1.19	0.99	0.94
HK2	Mean	4.12	3.08	3.96	3.72	3.32	3.56	3.40	3.43	2.88	2.88	3.24	3.72	3.88	3.32	3.64	3.12	4.20	3.16	3.53
	Std. Dev	1.27	1.12	1.14	0.55	1.18	1.33	1.32	0.60	1.05	1.20	1.33	1.06	1.24	0.58	1.08	1.51	0.96	1.03	0.57
YP1	Mean	5.36	5.18	5.09	5.21	4.86	5.00	5.18	5.02	4.68	4.64	5.00	4.95	5.32	4.92	5.32	5.09	5.36	5.45	5.31
	Std. Dev	0.73	0.85	1.06	0.62	1.21	1.20	0.96	1.02	1.25	1.18	1.11	1.21	0.84	1.01	0.72	0.97	0.66	0.74	0.62
YP2	Mean	4.85	4.38	4.12	4.45	4.42	4.62	4.50	4.51	3.81	4.00	4.73	3.88	4.46	4.12	4.85	4.54	4.88	4.69	4.74
	Std. Dev	0.78	0.90	1.24	0.73	1.06	1.06	1.07	0.87	1.44	1.23	0.83	1.42	0.99	0.65	0.83	1.07	0.59	0.84	0.63
PD1	Mean	4.65	5.00	5.12	4.92	4.31	4.65	4.69	4.55	4.81	4.73	4.77	4.62	4.81	4.75	4.85	4.65	4.58	4.92	4.75
	Std. Dev	0.85	1.02	1.03	0.74	1.19	1.47	1.12	1.08	1.20	1.43	1.03	1.30	1.13	0.98	1.29	1.09	1.33	1.02	1.01
PD2	Mean	4.29	4.29	4.46	4.35	4.38	4.58	4.46	4.47	4.42	4.25	4.63	4.17	4.54	4.40	4.50	4.33	4.63	4.92	4.59
	Std. Dev	0.95	0.91	1.28	0.84	1.10	1.14	1.18	1.02	1.14	1.48	1.17	1.13	1.06	0.99	1.14	1.09	1.17	0.97	0.90

**Table 5:** Park elements in relation to people’s restorative perceptions (*p* < 0.05).

		Being Away	Extent	Fascination	Compatibility
All ten research sites	Positive	Vegetation	Vegetation	Terrain	Vegetation, terrain
	Negative	Person	/	Person, rider, surrounding vehicles	Person, rider
Living and services	Positive	Vegetation	/	Vegetation,	Vegetation
	Negative	/	/	person, rider, Surrounding vehicles	/
Landscape and aesthetics	Positive	Vegetation, decorative lamp	Vegetation	Pavement, terrain	/
	Negative	/	/	/	/

**Table 6:** Correlation analysis results between people using behaviour and RCS (*p* < 0.05).

		B1	B2	B3	E1	E2	E3	F1	F2	F3	F4	F5	C1	C2	C3	C4	RCS
Frequency	Pearson correlation	−0.093	−0.025	0.026	−0.006	−0.008	0.06	0.131 *	0.092	−0.02	−0.051	−0.088	−0.128	−0.022	−0.157 *	0	−0.022
	.sig	0.157	0.705	0.698	0.923	0.908	0.362	0.047	0.164	0.758	0.436	0.18	0.052	0.74	0.017	0.998	0.734
Duration	Pearson correlation	0.242 **	0.169 **	0.07	0.185 **	0.125	0.144 *	−0.006	−0.007	0.146 *	0.170 **	0.192 **	0.237 **	0.180 **	0.222 **	0.148 *	0.213 **
	.sig	0	0.01	0.289	0.005	0.057	0.028	0.925	0.912	0.027	0.009	0.003	0	0.006	0.001	0.024	0.001

‘*’ and ’**’ indicates that indicators have significant influences.

## Data Availability

The data presented in this study are available on request from the corresponding author. The data are not publicly available due to the confidentiality of participants’ information.
